# The Erie County Hospital

**Published:** 1896-06

**Authors:** 


### THE ERIE COUNTY HOSPITAL.

AT THE time the JOURNAL goes to press, the smoke of battle has not yet cleared from the field of the county hospital contest sufficiently to disclose the features of the real victors. Enough has been learned, however, to call forth an expression of regret as the turn affairs seem to have taken.

The attitude of the JOURNAL during the whole discussion hat been plainly on the side of reform, as can be learned by referring to previous numbers. The editors of the present issue modestly venture the suggestion that the presence of one or two good, sensible, competent women to act as peacemakers might have saved—indeed may yet be the means of saving—a good deal of energy that is now expending itself without credit to anyone or any cause.

In this connection no harm will be done by the frank expression that some of the talking which has been done to conciliate "the galleries" has been unworthy of an enlightened and progressive profession. We refer to the soul-stirring descriptions of the enforced attendance at clinics on the part of patients as an invasion of their sacred liberties. Surely medical men ought to use every reasonable opportunity to inculcate the principle that to turn one's misfortune so as to prove of advantage to mankind is to act with praiseworthy self-sacrifice and altruism.

